# Study of a Fire-Resistant Plate Containing Fly Ashes Generated from Municipal Waste Incinerator: Fire and Mechanical Characteristics and Environmental Life Cycle Assessment

**DOI:** 10.3390/ma17081813

**Published:** 2024-04-15

**Authors:** Begoña Peceño, Yolanda Luna-Galiano, Fabiola Varela, Bernabé Alonso-Fariñas, Carlos Leiva

**Affiliations:** 1 Facultad de Ciencias del Mar, Escuela de Prevención de Riesgos y Medioambiente, Universidad Católica del Norte, Larrondo 1281, Coquimbo 1780000, Chile; begopc@ucn.cl (B.P.); fabiola.varela@ucn.cl (F.V.); 2 Departamento de Ingeniería Química y Ambiental, Escuela Técnica Superior de Ingeniería, Universidad de Sevilla, Camino de los Descubrimientos s/n, 41092 Seville, Spain; yluna@us.es (Y.L.-G.); bernabeaf@us.es (B.A.-F.)

**Keywords:** municipal solid waste incineration fly ashes, fire resistance, mechanical properties, environmental life cycle assessment

## Abstract

The recycling of fly ash from municipal solid waste incineration is currently a global issue. This work intends to examine the viability of a novel recycling alternative for fly ashes as a component of fire-resistant plates. To lessen the quantity of heavy metal leaching, the fly ash was utilized after being washed using a water/fly ash ratio of 2 for one hour. Subsequently, an inexpensive, straightforward molding and curing process was used to create a plate, with a composition of 60%wt of MSWI-FA, 30%wt of gypsum, 0.5%wt of glass fiber and 9.5%wt of vermiculite. The plate exhibited high fire resistance. Furthermore, it demonstrated compression, flexural strength and surface hardness slightly lower than the requirements of European Standards. This allows for manufacturing plates with a high washed MSWI-FA content as fire protection in firewalls and doors for homes and commercial buildings. A Life Cycle Assessment was carried out. The case study shows that a 60% substitution of gypsum resulted in an environmental impact reduction of 8–48% for all impact categories examined, except four categories impacts (marine eutrophication, human toxicity (cancer), human non-carcinogenic toxicity and water depletion, where it increased between 2 and 718 times), due to the previous washing of MSWI-FA. When these fly ashes are used as a raw material in fire-resistant materials, they may be recycled and offer environmental advantages over more conventional materials like gypsum.

## 1. Introduction

The yield of municipal solid waste (MSW) is large and presents a rapid growth rate. It is anticipated that the amount of MSW will more than double to 2200 million tons per year by 2025 [1]. Incineration not only reduces the amount and weight of solid waste but also produces energy and heat that may be utilized elsewhere. The most serious issue with incineration is the massive amount of waste produced by the process.

Municipal solid waste fly ashes (MSWI-FAs) are produced during municipal solid waste incineration. Due to the high concentration of heavy metals and the existence of soluble salts, MSWI-FAs are considered toxic. Three areas of MSWI-FA treatment have been identified for study: (a) heat processes; (b) solidification/stabilization methods; and (c) separation processes. Washing with water is one of the most used separating techniques [2,3,4]. The efficiency of unwanted metal leaching is greatly dependent on the ash pH, the ratio of water to solid (L/S) and the kind of metal. Various parameters, such as the L/S ratio and washing duration, have been researched in the literature [5], and it has been shown that the washing process may eliminate up to 90–95% of the chlorides while improving the consistency and quality of the fly ash for later recycling. In order to reduce the effluent, previous research suggested a 15 min duration and a 25 L/S ratio [6,7,8]. This essentially employed a 1 h L/S ratio of 2.

MSWI-FAs can be directly used as a Portland cement replacement [9,10,11] but they are commonly used with other raw materials in cement-making rotary kilns [12]. Another issue with MSWI-FAs high chloride level is that it might lead to corrosion and blockages. As a result, it is critical that MSWI-FAs are properly pre-treated before being utilized as a raw material. Previous studies have analyzed the use of MWSI-FA in CO_2_ capture [13], alkali-activated cements [14], concrete [15], glass ceramics [16], bricks [17], asphalts [18], and in paste backfill in underground mine workings [19].

The fire resistance of a construction material is a characteristic determined by the ability of a product to maintain its properties under the incidence of fire for a limited time [20]. The fire resistance of construction elements is evaluated under three main criteria: (a) stability or integrity, (b) no emission of flammable gases and (c) thermal insulation. Thermal insulation is very important for the construction of fire-safe environments, and it is defined as the time delay before reaching a certain critical temperature, 180 °C in the non-exposed fire in the case of a plate exposed on one side to a fire.

Similar to MSWI-FA in terms of chemical composition and qualities, certain commercial products are utilized as thermal insulation and/or passive fire protection. Numerous references to fire-resistant materials can be found in many study results. These materials include varying amounts of different types of combustion fly and bottom ashes [21,22], coal [23], biomass [24], ladle [25] and granulated blast furnace [26], slags, seashells [27] and waste from construction and demolition [28].

A plate composed primarily of MSWI-FA after washing and manufactured utilizing straightforward and inexpensive techniques has been made, with adequate fire and mechanical properties and a low environmental impact. For this last goal, a Life Cycle Assessment was carried out, comparing a product composed of gypsum, vermiculite and fiber with another replacing 60%wt of the gypsum with washed MSWI-FA. The main industrial application of the material presented here is for fire-resistant partitioning elements, such as barriers and doors, in installations within buildings; this kind of material can be used in place of commercial fire-resistant materials made of gypsum or calcium silicate.

## 2. Materials and Methods

### 2.1. Materials

Regarding this research, fly ash from a single plant from a metropolitan area in Spain is used. The feed stream is mostly made up of residential waste, with a tiny contribution from commercial sources. The chemical compositions of MSWI-FA can be seen in Table 1.

The main elements in MSWI-FA are calcium and silica. The loss on ignition of MSWI-FA is very high, which indicates the presence of large amounts of unburned carbonaceous materials and carbonates. According to EN 1097-7 [29], MSWI-FA has a specific gravity that is 29% lower than that of commercial gypsum (4.1 g/cm^3^). When it comes to heavy metals, Pb and Zn are the most concentrated in fly ash because they are carried by the stream as volatile chlorides that subsequently condense on the MSWI-FA particles.

**Figure 1:**
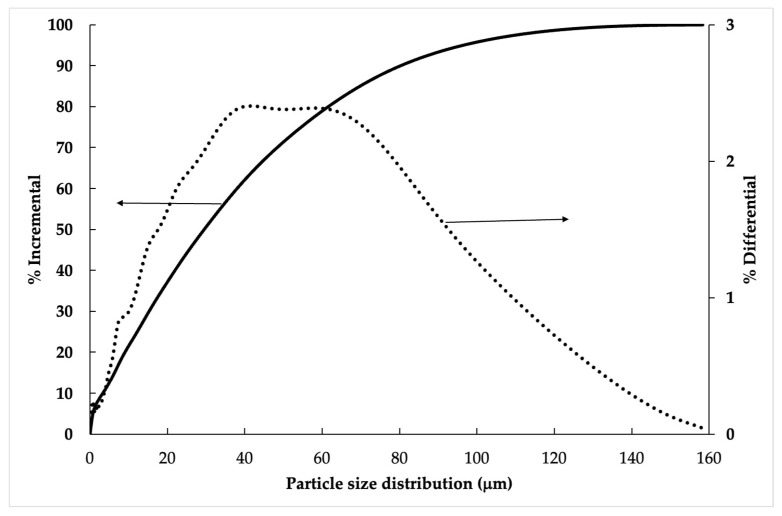
Particle size distribution of MSWI-FA.

Particle size distribution of the MSWI-FA is small. MSWI-FA showed particle sizes of 15–150 μm, measured by sieving, with a D50 of 30 μm, as seen in Figure 1. This was measured using a Saturn DigiSizer II Particle Size Analyzer.

The X-ray pattern of these ashes indicates that the predominant phases are halite (NaCl) and sylvite (KCl), with the minor phases being lime, calcium sulphate and calcium carbonate [3,30].

In the literature [1,5,7,8,31], numerous parameters, including time and the liquid-to-solid (L/S) ratio, have been investigated. Different L/S ratios between 1 and 25 have been analyzed, with contact times between 15 min and 2 h. Some of them establish an optimal L/S between 2 and 3, although this depends on the particle size, pH, and type of heavy metals of the MSWI-FA. In this work, MSWI-FA was washed with L/S ratio of 2 to reduce the number of heavy metals of MSWI-FA with a low L/S ratio. The MSWI-FA and water mixtures were put in contact in an orbital shaker for 1 h at 20 °C. The liquid and solid phases were separated by vacuum filtration and further dried in ambient conditions (20 °C; average relative humidity: 45%) for 60 days. More than 90% of the Cl and more than 50% of the Na and K can be removed in these washing conditions [32].

Gypsum (G) was used as the binder in accordance with EN 13279-1:2009 [33]. As an addition, vermiculite (V) was used. Vermiculite is a hydrated silicate with a flaky structure that contains iron, magnesium and aluminum. Vermiculite is typically added to mortars used for fire protection, as per earlier research [34,35]. The vermiculite from VERLITE company (Gijón, Spain) presents an 84.9% particle size lower than 1.4 mm in size. To strengthen the building material’s mechanical resistance to bending and fissuring, glass fibers with a length of 2–4 cm and a diameter of 25 µm were employed (tensile strength 2400 MPa and Young’s modulus of 80.0 GPa).

Furthermore, a comparison and testing were conducted on two commercially made plates intended for passive fire protection. Commercial 1 was a calcium silicate-based plate, and Commercial 2 was a gypsum-based plate with vermiculite (9.5%wt) and glass fibers (0.5%wt).

### 2.2. Plate Manufacturing

The objective was to develop a plate mostly made of MSWI ash. The plate composition was consistent with previous studies [35], keeping two primary goals in mind: first, the overwhelming component (>50%wt) must be made up of ashes, and, second, the products must have a minimum set of mechanical qualities determined by the intended usage.

A planetary mixer was filled with the solid ingredients listed in the preceding table, and the mixture was stirred until it was homogenous. After that, water was added to the mixture and stirred once more to create a homogenous paste. The obtained paste was poured into molds. After a day, they were removed from the molds and allowed to cure for over 27 days at room temperature (average temperature: 20 °C; average relative humidity: 45%) before being subjected to different tests.

### 2.3. Thermal Insulating Behaviour

The common fire resistance test is described in European regulation EN 1363-1 [36], which is equivalent to other widely used international regulations. According to the test, one side of the fire-resistant material must be subjected to heat in accordance with the equation T = 20 + 345log10(8t + 1), where t is the amount of time in minutes since the test began and T is the fire temperature in degrees Celsius.

Utilizing an S-type thermocouple positioned within the oven, we recorded the temperature of the exposed face of the plate (hot surface, T_in_) in this furnace. Subsequently, we employed the thermocouple to fine-tune the oven temperature through a proportional controller, thereby achieving the generation of a standard temperature curve. The temperature was measured on the unexposed face (cold surface, T_out_) using a Pt-100 temperature sensor. A plate of 40 cm in height, 18 cm in breadth and 2 cm in thickness was tested.

The time required for T_out_ to reach 180 °C (t_180_) is regarded as a standard value for investigating thermal insulating capacity by EN 1363-1.

### 2.4. Physical and Mechanical Properties

The bulk density (kg/m^3^) of the plate was determined as the ratio of the dry mass and its volume. A thermogravimetric study was performed to assess the mass change with temperature. The sample was heated at a rate of 20 °C per minute in an air environment and examined using measurements from a TGA/SDTA 851 thermogravimetric analyzer (Metter-Toledo, Barcelona, Spain) [27]. The measurement was made in a temperature range from 20 °C to 1000 °C.

The pH was measured according to European standard, EN 12859 [37]. Therefore, 2 g of the material was dissolved in 20 g of agitated water, and the pH of the solution was determined after 5 min. The water absorption capacity (A) was tested in accordance with European standard EN 12859 [37]. After measuring the mass of the board before immersing it in water (M1), the water temperature must be 23 °C ± 3 °C. After 120 min, the plate is removed from the water, samples are allowed to drain for 5 min and the board’s weight (M2) is measured. A is determined using:

A = 100 · (M2 − M1)/M1(1)

A machine (Suzpecar, MEM-102/50 t) was also used to assess the compressive (R_c_) and flexural (R_f_) [38] strengths. Flexural strength tests were performed on three 16 cm × 4 cm × 4 cm samples, as well as compressive strength tests on six samples following flexural strength. The loading rate was controlled by the displacement of the top face, which was tensioned at a rate of 0.5 mm/min. Because some building materials may be susceptible to impact, the material’s surface hardness (D) according to EN 12859 [37] was measured using a Shore C durometer. For the repeat experiment, two identical plates measuring 40 × 18 cm in surface area and 2 cm in thickness were utilized.

### 2.5. Environmental Life Cycle Analysis

To assess the possible diminution in environmental impacts achieved by partially replacing MSW ash with gypsum, a comparative Life Cycle Assessment (LCA) was conducted. The LCA methodology was implemented in compliance with ISO 14044 guidelines [39].

#### 2.5.1. Scope and Goal

**Figure 2:**
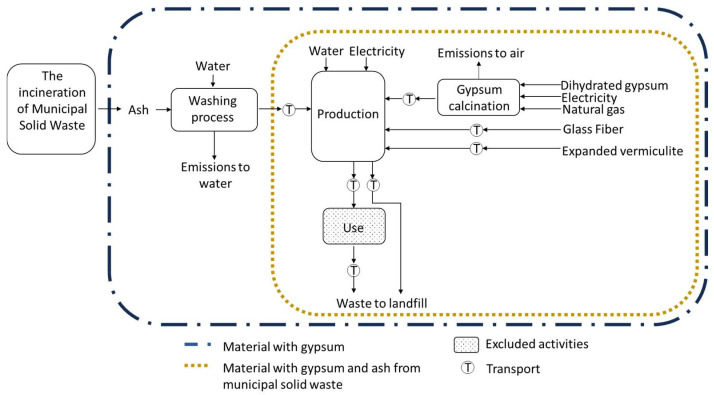
System boundaries for the life cycle of fire resistance material with municipal solid waste ash.

This study’s primary objective was to evaluate the innovative fire-resistant material’s environmental sustainability using MSWI-FA. The scope was from “cradle to gravel”. The main stages and the foreground model for traditional materials (commercial-2) and replacing 60% of gypsum with MSWI-FA (P-MSWI-FA) systems used in LCA are shown in Figure 2.

According to EN standard 1363-1 [36], the functional unit is defined as the material that must satisfy the same fire resistance for both plates (P-MSWI-FA and Commercial 2). Thus, the thickness of the plate is determined after obtaining the fire resistance results (Section 3.2. Thermal Insulating capacity). This is crucial because the ultimate durations of fire resistance are greatly impacted by the parameter of material thickness [21]. The plates’ other measurements—height and length—were set at one meter.

#### 2.5.2. Life Cycle Inventory and Sensitivity Analysis

The thickness of the plates was determined, and the Section 3 includes foreground inventory data expressed per functional unit (a surface area of 1 m^2^ and the required thickness for both materials to possess the same fire resistance properties). The background inventory data for all of the inputs and outputs were condensed into Ecoinvent 3.10 [40].

With respect to raw materials, the gypsum hemihydrate was obtained from the calcination of dehydrated gypsum. Dehydrated gypsum was dried using a rotary kiln, which used electricity and natural gas for the process. Per ton of gypsum hemihydrate produced, the energy consumption was 28 kWh of electricity and 820.6 MJ of thermal energy (losses included) [41]. During dehydration, the number of emissions from burning natural gas was determined using Labein [42]. MSWI-FAs were obtained from the incineration of municipal solid waste. Following this, and to lower the concentration of heavy metals, the MSWI-FA was cleaned using an L/S ratio of 2. Emissions from the washing process were determined according to Phua et al. [30]. Glass fiber and vermiculite were acquired from Ecoinvent 3.10 [40].

With respect to production, as shown in Figure 1, the vermiculite, glass fiber and gypsum were mixed. For commercial 2, the glass fiber and vermiculite had the same composition as shown in Table 2, and the commercial gypsum had a composition of 90%. And the relationship between water and solid was 0.45 for 100% gypsum [43]. The density of commercial 2 for this composition was defined according to Gencel et al. [43]. For P-MSWI-FA, the compositions of vermiculite, glass fiber, gypsum, fly ashes and water are shown in Table 2, and the density is provided in the results of physical and mechanical properties. Subsequently, operational data of fire-resistant materials, such as electricity consumption and the moisture content of the fire resistance materials, were obtained. The material was lost during demoulding, and the rejected material of the final product by quality controls is defined according to Peceño et al. [27].

In terms of waste management, waste from use and manufacturing was dumped in landfills and categorized as solid demolition waste under 2000/532/EC (European Commission, 2000) [44].

In terms of transport, the production plant of fire resistance plates was assumed to be in the production plant in Lleida (Catalonia, NE Spain). Both the closest gypsum quarry and gypsum calcination plant to production plant were in Gelsa (291 km from production plant). The additives of material were assumed in the vermiculite plant of Verlite in Gijon (Asturias, N Spain) to 835 km from production plant and glass fiber plant of zero composite in Velez (Andalucía, S Spain) to 1014 km from production plant. These companies manufacture and commercialize these products. The MSWI-FA was assumed in the urban waste incineration plant in Tarragona (Catalonia, NE Spain) to be 145 km from the production plant. The production plant’s waste was transported 155 km to the closest landfill. It was anticipated that fire resistance materials would travel 200 km to their intended use after being produced. End-of-life-generated waste was thought to be transported to the landfill 100 km away [45]. Distances were calculated in relation to transportation activities using the Google MapsTM application [46]. All transportation was conducted by road, and in accordance with the classification found in Regulation (EC) No. 715 [47], a 16–32-ton Euro 6 truck was utilized.

A sensitivity analysis was performed to determine the robustness of the findings. Following the analysis, important parameters of the plate were covered, which could influence environmental scenarios among the materials.

#### 2.5.3. Environmental Impact Assessment

The life cycle modeling utilized the Sigma-Pro version 9.5.0 LCA software developed by PRé Consultants [48]. Environmental impact calculations were performed using the Recipe 2016 (midpoint) methodology, as outlined by Huijbregts et al. [49]. All impact categories provided by Recipe 2016 (midpoint) were examined, encompassing ozone depletion (ODP); freshwater eutrophication (FEP); terrestrial acidification (TAP); marine eutrophication (MEP); photochemical oxidant formation: ecosystem (EOFP); human toxicity: non-cancer (HTPnc); fine particulate matter formation (PMFP); human toxicity: cancer (HTPc); terrestrial ecotoxicity (TETP); photochemical oxidant formation: human health (HOFP); water depletion (WCP); land use (LOP); mineral resource scarcity (SOP); marine ecotoxicity (METP); fossil resource scarcity (FFP); climate change (GWP); and freshwater ecotoxicity (FETP).

## 3. Results and Discussion

### 3.1. Physical Properties

**Figure 3:**
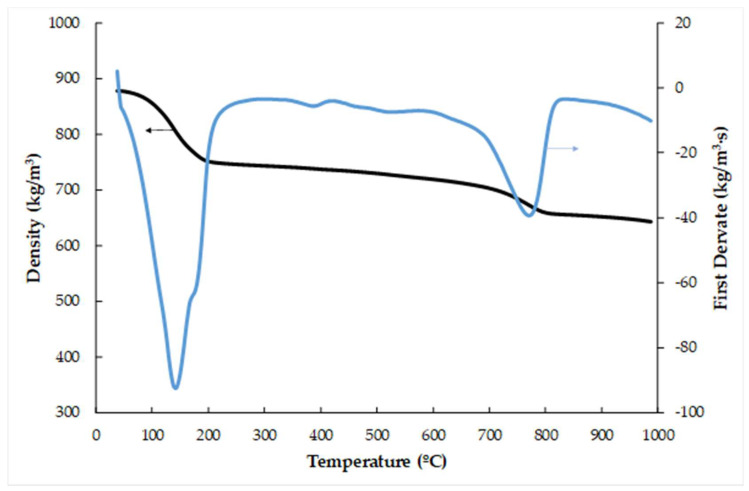
TG and SDTA curves of the P-MSWI-FA.

The density of P-MSWI-FA is 877 kg/m^3^, which is very low, and it is compared with other plates with wastes due to the vermiculite effect [36] and lower than a gypsum plate with the same composition (1150 kg/m^3^) due to the lower specific density of MSWI-FA (Table 1) compared with gypsum-specific density (4.1). Gypsum-based materials are categorized into three groups according to EN 12859 [37]: high (1100 ÷ 1500 kg/m^3^), medium (800 ÷ 1100 kg/m^3^) and low (600 ÷ 800 kg/m^3^). P-MSWI-FA falls within the medium-density category. The variation in the density with the temperature is an important factor to analyze to observe the behavior during the thermal insulating test, and it can be seen in Figure 3. The density remains almost constant up to 70 °C. There is a decrease in density between 70 and 220 °C. Between 70 and 105 °C corresponds to the release of free water (3.1%). Between 105 and 220 °C, the loss density is due to the chemically bound water in the form of CaSO_4_·2H_2_O (10.7%), both that provided by the commercial gypsum (10.4 × 0.3 = 3.1%) and that provided by MSWI-FA (10.7 − 3.1 = 7.6%), which decomposes endothermically, producing CaSO_4_ and releasing H_2_O(g) [24]. The material loses weight again between 220 and 600 °C because the crystalline water in the vermiculite releases during this time, resulting in a 4% drop in weight [34]. The CaCO_3_ in MSWI-FA undergoes endothermic breakdown into CaO and CO_2_ (g) in a temperature range of 650 °C to 800 °C (7%), while the unburned material included in the MSWI ashes starts to burn at temperatures higher than 900 °C.

A pH value of 8.6 for P-MSWI-FA was determined, which allows this plate to be classified as a normal plate according to EN 12859 [37] because its pH is between 6.5 and 10.5. Although MSWI-FA presents a high pH, the effect of the washing process diminishes the pH. The water absorption of the plate is 26.3%, a very high value, due to the high porosity produced by the vermiculite [35], which allows for greater absorption of water.

### 3.2. Thermal Insulating Capacity

**Figure 4:**
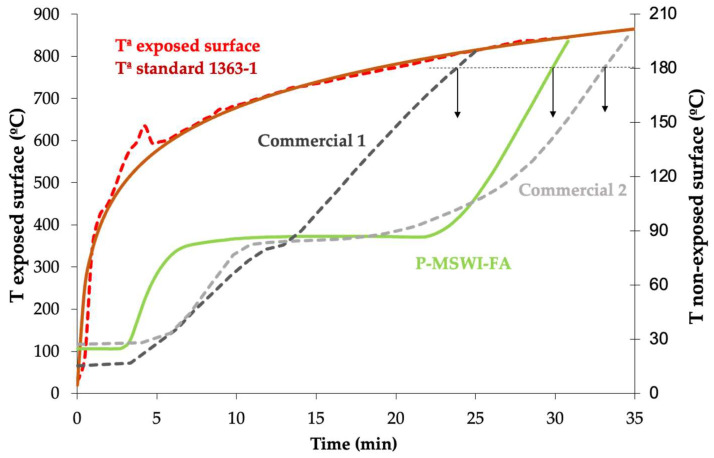
Thermal insulation of the materials.

By establishing the amount of time required for the non-exposed side to attain a temperature of 180 °C (t180) when the exposed side is subjected to the specified temperature–time, the insulating capacity of the P-MSWI-FA and two commercial plates (commercial 1 and 2) was determined. Figure 4 shows the results for the thermal test for 20 mm thick plates. P-MSWI-FA presents a t180 of 29.9 min. This outcome is far superior to 23 min for the commercial plate made of calcium silicate (Commercial 1) and slightly lower (9%) than for a plate composed of gypsum, fibers and vermiculite (Commercial 2), using the same oven and plate thickness. The product under test has a higher insulating ability than plates with the same dimensions that are created with a percentage similar to that of the product in this study for biomass ashes [24] and fly ash from coal combustion [35].

Water can be present in fire-resistant materials in a variety of forms and concentrations, including free, adsorbed and/or crystallized water. An overpressure develops inside the paste when this water evaporates as a result of a heating action, such as fire. This overpressure causes the steam to be directed toward the cylinder’s interior zones, where it cools and condenses once again since these regions are colder. Consequently, a liquid layer is formed and is transferred to the non-exposed surface, with a minor displacement. The temperature on the non-exposed surface is kept at around 100 °C during this phase since the fire energy is being used up. As seen in Figure 4 [24], this action causes an evaporation plateau to form, which is a time when the temperature of the non-exposed surface remains constant. Initially, P-MSWI-FA grows very quickly, due to its lower density, and heats up more quickly than the two commercial products; however, P-MSWI-FA has a long-lasting evaporation plateau, even longer than commercial 2, because the energy absorbed by the calcium sulfate and calcium carbonate present in MSWI-FA is greater.

**Figure 5:**
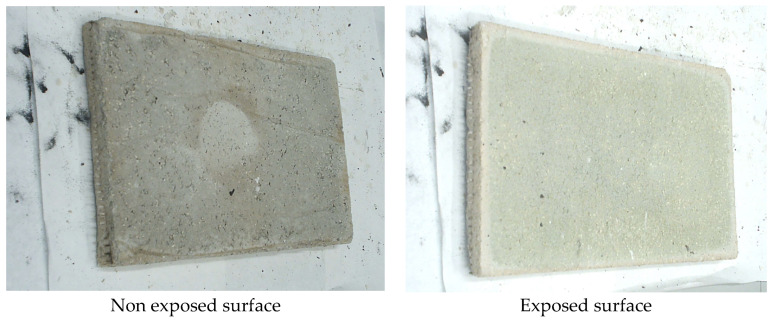
Exposed and non-exposed surface after the fire test.

Lastly, it should be mentioned that throughout the test, the plate did not release any smoke. It showed stability throughout the test, maintained integrity and was free of cracks during the fire test, as shown in Figure 5.

### 3.3. Mechanical Properties

Table 3 shows the compressive (Rc) and flexural strengths (Rf) and the surface hardness (D).

EN 13279-1 standard [33] establishes 1 MPa as the minimum compressive strength of a plate, which is higher than the value of P-MSWI-FA. This is due to the fact that MSWI-FA contains gypsum, but after the washing process, it becomes CaSO_4_·2H_2_O and, thus, does not set or provide mechanical resistance during the plate manufacturing process. The plate has a lower compressive strength than other materials with various residues [35,50] but higher than plates with gasification biomass ash [51], probably due to both fly ashes presenting a high LOI.

The material’s surface hardness was 36 Shore C units. The standard EN 12859 [37] specifies a minimum surface hardness of 55 Shore C for medium-density plates. This result is significantly less than the hardness of several gypsum plates (ranging from 45 to 70 Shore C units) but higher than plates with gasification biomass ash [51], probably due to both ashes presenting a high LOI and low specific gravity, producing a low-density plate.

### 3.4. Environmental Life Cycle Analysis

According to the results in Section 3.2, the commercial panel must have a thickness of 1.8 cm (9% less than the P-MSWI-FA plate). Based on this, Table 4 displays the life cycle inventory.

**Figure 6:**
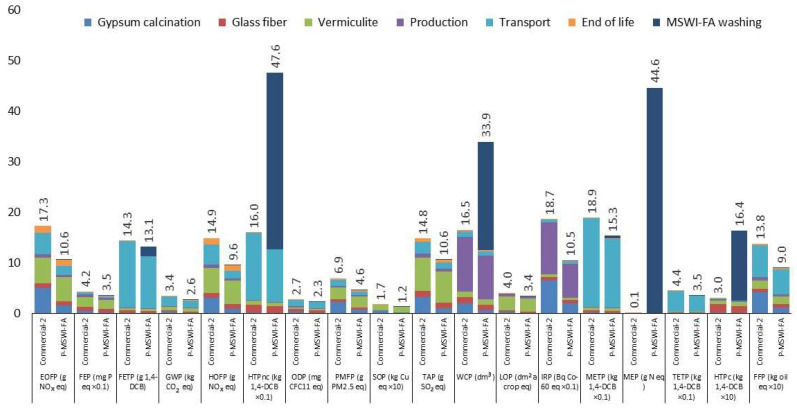
Environmental effects of a single square meter of both types of fireproofing materials. Note: A few impacts have been adjusted for fit. Multiply the initial value by the relevant impact factor displayed on the *x*-axis to obtain the original value.

The environmental effects of both materials life cycles (commercial 2 and P-MSWI-FA) are displayed in Figure 6.

As depicted in Figure 6, introducing MSW-FA waste led to a decrease in 14 out of 18 impacts (HTPnc—human toxicity: non-cancer; WCP—water depletion; MEP—marine eutrophication; HTPc—human toxicity: cancer). The increase in HTPnc, WCP, MEP and HTPc is mainly due to water consumption in the washing process of MSWI-FA and heavy metal and nitrogen emissions.

The transportation of raw materials is the primary contributor to 8 out of the 18 impacts (GWP—climate change; ODP—ozone depletion; TETP—terrestrial ecotoxicity; FETP—FETP: freshwater ecotoxicity; MEP, METP—marine ecotoxicity, HTPnc; FFP—fossil resource scarcity) for the case of commercial 2. This is attributed to the consumption of fossil fuels. Regarding impacts related to toxicity (HTPnc, TETP, METP, FETP), they are attributed to emissions associated with the use of brakes and tires during transportation. The same trend occurred for P-MSW-FA, except for the impact categories HTPnc and HTPc, where the main contributor was MSW-FA washing.

In the production, the consumption of water and electricity was the main contributor in the IRP (55%) and WCP (65%) impact categories, respectively, for the case of commercial 2. Similarly, for P-MSW-FA, production caused 64% of the IRP impact category, and in the case of WCP impact, production accounted for 26% of the impact, behind MSW-FA washing.

Both for commercial 2 and P-MSW-FA, vermiculite was the main contributor in 7 out of the 18 impacts (HOFP—photochemical oxidant formation: human health; PMFP, EOFP—photochemical oxidant formation: ecosystem; TAP—terrestrial acidification; FEP—freshwater eutrophication; LOP—land use; SOP—mineral resource scarcity). The consumption of fossil fuel during the thermal process for generating expanded vermiculite and the use of land during vermiculite extraction contributed to more than 30% in seven impact categories.

For commercial 2, the emissions of As and Cr (VI) associated with the fiberglass manufacturing process caused 52% of the HTPc impact category. However, for P-MSW-FA, fiberglass had a contribution of less than 20% (ODP).

In both materials, gypsum contributed less than 35%. The decrease in gypsum consumption in P-MSW-FA resulted in its contribution being present in only 6 out of the 18 impact categories (IRP, PMFP—fine particulate matter formation; EOFP, HOFP, SOP and FFP), with a contribution ranging between 20% and 10% of the total impact. However, in the case of commercial 2, gypsum had a significant contribution (between 10% and 35%) in 11 out of the 18 impacts.

#### Sensitivity Analysis

**Figure 7:**
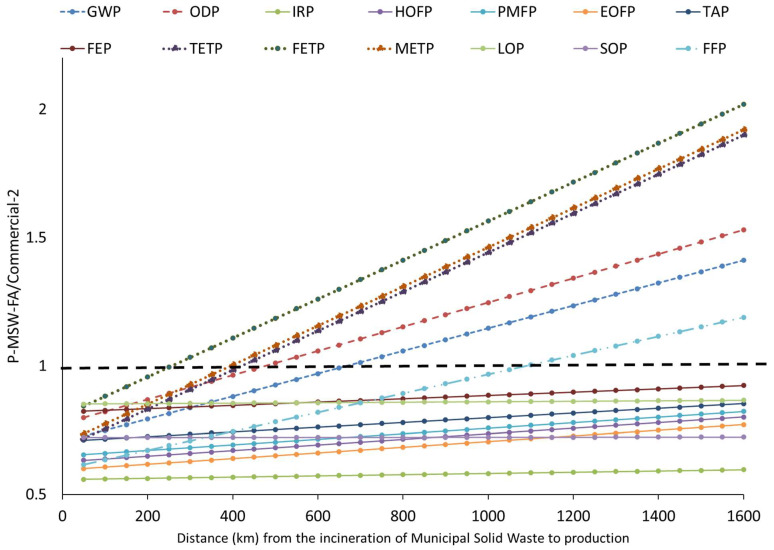
The environmental impact of the distance from municipal solid waste incineration to production. Refer to Figure 6 for details on the nomenclature’s significance.

The transportation of raw materials is a process that contributes significantly to the environmental impacts. As shown in Figure 7, when the distance from municipal solid waste incineration to production was modified, it was observed that six impact categories (METP, FETP, TETP, GWP, FFP and ODP) underwent changes in their scenarios. When the distance increases from 150 km to 300 km compared to the original distance (145 km), four impact categories would face worse scenarios (FETP, TETP, METP). This is because in P-MSW-FA, the contribution of transportation to the total impact is greater than 75%. When the distance increases between 350 and 600 km compared to the original distance, the P-MSW-Fa material exhibits a worse environmental scenario in the GWP and ODP impact categories. Finally, when the distance increases by 900 km, the FFP impact category presents a worse scenario. For the impact categories WCP, HTPc, HTPnc and MEP, the P-MSW-FA material would still present a worse scenario compared to the commercial one when any distance of the raw materials is modified.

As shown in Supplementary Figures S1–S3 in the Supplementary Information, the variation in the distance from gypsum calcination, fiberglass, and vermiculite to production, respectively, did not lead to changes in the impacts of the P-MSW-Fa material compared to the commercial 2 material.

## 4. Conclusions

This new eco-friendly material, composed of 60% fly ash from municipal solid waste, shows diminished mechanical and physical properties. According to European standards, it has slightly lower mechanical properties. Our results suggest that the ash compositions should be reduced to achieve technically feasible properties (up to 50%). In terms of physical characteristics, it exhibits lower density compared to the material produced with gypsum when substituting 60% of municipal waste fly ash.

Regarding the thermal insulating capacity, the developed material with 60% fly ash demonstrates superior insulation, with a time of 29.9 min to reach 180 °C, surpassing calcium silicate and slightly trailing gypsum with fibers and vermiculites.

In comparison to conventional material (gypsum), recycling fly ash from municipal waste incineration in fire-resistant materials can have positive environmental effects, according to the environmental Life Cycle Assessment. However, the washing process and wastewater purification process must be optimized to reduce the impacts of carcinogenic human toxicity, marine eutrophication, non-carcinogenic human toxicity and water depletion.

## Figures and Tables

**Table 1:** MSWI-FA chemical composition (%wt).

Parameter	%wt
Specific gravity	2.89
Moisture	3.04
Loss on ignition	18.42
CaO	37.06
MgO	1.37
Fe_2_O_3_	0.86
Al_2_O_3_	4.72
SiO_2_	6.56
K_2_O	4.45
Na_2_O	6.72
Cl	12.17
SO_3_	3.15
MgO	1.29
ZnO	1.41
P_2_O_5_	0.76
PbO	0.45
TiO_2_	0.46
SnO_2_	0.15

**Table 2:** Composition (weight%) of the paste.

Material	Water/Solids Ratio	Glass Fiber (%wt)	Vermiculite (%wt)	Gypsum (%wt)	Ash (%wt)
P-MSWI-FA	0.4	0.5	9.5	30	60

**Table 3:** Mechanical properties of the plate.

Parameter	Compressive Strength (Rc, MPa)	Flexural Strength (Rf, MPa)	Superficial Hardness (D, Shore C)
P-MSWI-FA	0.84 ± 0.2	0.73 ± 0.1	36 ± 0.6

**Table 4:** Inventory data for a 1 m^2^ surface and the required thickness for both materials to possess the same fire resistance properties.

Section	Input Flow	Output Flow	Unit	Commercial 2	P-MSWI-FA
MSW-FA washing	MSW-FA		G	-	11.11
	Water		Kg	-	22.21
		Emission to water			
		As	Mg		77.76
		Ba	G		3.00
		Cd	Mg		2.22
		Cr	G		0.15
		Cu	Mg		17.78
		Mo	G		1.39
		Ni	Mg		45.54
		Pb	G		2.05
		Sb	Mg		9.45
		Se	G		0.12
		Zn	G		0.30
		Hg	Mg		2.17
		N	G		150.00
Gypsum Calcination	Gypsum dihydrate		Kg	20.87	6.59
	Natural gas		MJ	14.44	4.02
	Electricity		kWh	0.58	0.18
		Emissions to air			
		CH_4_	Mg	20.21	6.38
		CO	Mg	144.39	45.57
		CO_2_	Mg	805.68	254.28
		VOC	Mg	72.19	22.79
		NOx	Mg	750.82	236.97
		NO_2_	Mg	14.44	4.56
Raw Materials	Gypsum hemihydrate ^1^		Kg	17.60	5.55
	Vermiculite		Kg	1.86	1.76
	Fiberglass		Kg	0.10	0.09
Production	Water		Kg	8.75	7.37
	Electricity		kWh	0.97	0.63
		Waste to Landfill	Kg	2.80	2.42
End of life		Waste to Landfill	Kg	20.70	17.74
Transport	MSWI -FA to Production		Tkm	-	1.61
	Calcination to Production		Tkm	5.12	1.62
	Vermiculite manufacturer to production		Tkm	1.55	1.47
	Fiberglass manufacturer to production		Tkm	0.10	0.09
	Production to landfill		Tkm	0.43	0.37
	Production to use		Tkm	4.14	3.55
	Use to landfill		Tkm	2.07	1.77

^1^ Intermedium stream to production (included in the table just in terms of balance).

## Data Availability

All data used to support the findings of this study are available from the corresponding author upon request.

## Supplementary Materials

The following supporting information can be downloaded at: https://www.mdpi.com/article/10.3390/ma17081813/s1, Figure S1: The environmental impact of the distance from gypsum calcination to production. Refer to Figure 6 for details on the nomenclature’s significance; Figure S2: The environmental impact of the distance from vermiculite plant to production. Refer to Figure 6 for details on the nomenclature’s significance; Figure S3:The environmental impact of the distance from glass fiber plant to production. Refer to Figure 6 for details on the nomenclature’s significance.

## References

[1] Chen D., Zhang Y., Xu Y., Nie Q., Yang Z., Sheng W., Qian G. (2022). Municipal solid waste incineration residues recycled for typical construction materials. RSC Adv..

[2] Mangialardi T. (2001). Sintering of MSW fly ash for reuse as a concrete aggregate. J. Hazard. Mater..

[3] Chimenos J., Fernández A., Cervantes A., Miralles L., Fernández M., Espiell F. (2005). Optimizing the APC residue washing process to minimize the release of chloride and heavy metals. Waste Manag..

[4] Chen W., Kikelund G.M., Jensen P.E., Ottosen L.M. (2017). Comparison of different MSWI fly ash treatment processes on the termal behavior of As, Cr, Pb, and Zn in the ash. Waste Manag..

[5] Maresca A., Bisinella V., Astrup T.F. (2022). Life cycle assessment of air-pollution-control residues from waste incineration in Europe: Importance of composition, technology and long-term leaching. Waste Manag..

[6] Chen X., Bi Y., Zhang H., Wang J. (2016). Chlorides removal and control through water-washing process on MSWI fly ash. Procedia Environ. Sci..

[7] Mangialardi T. (2003). Disposal of MSWI fly ash through a combined washing-immobilization process. J. Hazard. Mater..

[8] Wang Q., Yang J., Wang Q., Wu T. (2009). Effects of water-washing pretreatment on bioleaching of heavy metals from municipal solid waste incinerator fly ash. J. Hazard. Mater..

[9] Poranek N., Pizoń J., Łaźniewska-Piekarczyk B., Czajkowski A., Lagashkin R. (2024). Recycle Option for municipal Solid Waste Incineration Fly Ash (MSWIFA) as a Partial Replacement for Cement in Mortars Containing Calcium Sulfoaluminate Cement (CSA) and Portland Cement to Save the Environment and Natural Resources. Materials.

[10] Marieta C., Martín-Garin A., Leon I., Guerrero A. (2023). Municipal Solid Waste Incineration Fly Ash: From Waste to Cement Manufacturing Resource. Materials.

[11] Rémond S., Pimienta P., Bentz D.P. (2002). Effects of the incorporation of Municipal Solid Waste Incineration fly ash in cement pastes and mortars: I. Experimental study. Cem. Concr. Res..

[12] Joseph A.M., Snellings R., Van den Heede P., Matthys S., De Belie N. (2018). The Use of Municipal Solid Waste Incineration Ash in Various Building Materials: A Belgian Point of View. Materials.

[13] Mokrzycki J., Baran P., Gazda-Grzywacz M., Bator J., Wróbel W., Zarębska K. (2023). Decarbonatization of Energy Sector by CO_2_ Sequestration in Waste Incineration Fly Ash and Its Utilization as Raw Material for Alkali Activation. Materials.

[14] Liu J., Wang Z., Xie G., Li Z., Fan X., Zhang W., Xing F., Tang L., Ren J. (2022). Resource utilization of municipal solid waste incineration fly ash cement and alkali-activated cementitious materials: A review. Sci. Total Environ..

[15] Cheng Y., Dong Y., Diao J., Zhang G., Chen C., Wu D. (2019). MSWI Bottom Ash Application to Resist Sulfate Attack on Concrete. Appl. Sci..

[16] Liu B., Yang Q.W., Zhang S.G. (2019). Integrated utilization of municipal solid waste incineration fly ash and bottom ash for preparation of foam glass-ceramics. Rare Met..

[17] Lin T.-H., Siao H.-J., Gau S.-H., Kuo J.-H., Li M.-G., Sun C.-J. (2023). Life-Cycle Assessment of Municipal Solid Waste Incineration Fly Ash Recycling as a Feedstock for Brick Manufacturing. Sustainability.

[18] Li E., Zhang X., Wang L., Wang R., Zhang W., Chen C., Zhang W. (2023). Performance of Asphalt Mastic and Asphalt Mixture with Harmless Municipal Solid Waste Incineration Fly Ash. Buildings.

[19] Korzeniowski W., Poborska-Mynarska K., Skzypkowski  K. (2018). The idea of the recovery of municipal solid waste incineration (MSWI) residues in Klodawa salt mine S.A by filling the excavations with self-solidifying mixtures. Arch. Min. Sci..

[20] (2019). Fire Classification of Construction Products and Building Elements Part 1: Classification Using Data from Reaction to Fire Tests.

[21] Vilches L.F., Leiva C., Vale J., Fernández-Pereira C. (2005). Insulating capacity of fly ash pastes used for passive protection against fire. Cem. Concr. Compos..

[22] García Arenas C., Marrero M., Leiva C., Solís-Guzmán J., Vilches Arenas L.F. (2011). High fire resistance in blocks containing coal combustion fly ashes and bottom ash. Waste Manag..

[23] Peceño B., Pérez-Soriano E.M., Ríos J.D., Luna Y., Cifuentes H., Leiva C. (2023). Effect of different ashes from biomass olive pomace on the mechanical and fire properties of gypsum-based materials. Rev. Construcción.

[24] Leiva C., Vilches L.F., Vale J., Fernández-Pereira C. (2009). Fire resistance of biomass ash panels used for internal partitions in buildings. Fire Saf. J..

[25] Peceño B., Pérez-Soriano E.M., Luna-Galiano Y., Leiva C. (2023). The Incorporation of Ladle Furnace Slag in Fire Insulating Gypsum-Based Materials. Fire.

[26] Ríos J.D., Arenas C., Cifuentes H., Vilches L.F., Leiva C. (2020). Development of a paste for passive fire protection mainly composed of granulated blast furnace slag. Environ. Prog. Sustain. Energy.

[27] Peceño B., Alonso-Fariñas B., Vilches L.F., Leiva C. (2021). Study of seashell waste recycling in fireproofing material: Technical, environmental, and economic assessment. Sci. Total Environ..

[28] Leiva C., Solís-Guzmán J., Marrero M., García Arenas C. (2013). Recycled blocks with improved sound and fire insulation containing construction and demolition waste. Waste Manag..

[29] (2009). Tests for Mechanical and Physical Properties of Aggregates Part 7: Determination of the Particle Density of Filler Pyknometer Method.

[30] Ontiveros J.L., Clapp T.L., Kosson D.S. (1989). Physical properties and chemical species distributions within municipal waste combuster ashes. Environ. Prog..

[31] Phua Z., Giannis A., Dong Z.L., Lisak G., Jern Ng W. (2019). Characteristics of incineration ash for sustainable treatment and reutilization. Environ. Sci. Pollut. Res..

[32] Wang K.S., Chiang K.Y., Lin K.L., Sun C.J. (2001). Effects of a water-extraction process on heavy metal behavior in municipal solid waste incinerator fly ash. Hydrometallurgy.

[33] (2008). Gypsum Binders and Gypsum Plasters Part 1: Definitions and Requirements.

[34] Vilches L.F., Leiva C., Olivares J., Vale J., Fernández C. (2005). Coal fly ash-containing sprayed mortar for passive fire protection of steel sections. Mater. De Construcción.

[35] Leiva C., Arenas C., Vilches L.F., Alonso-Fariñas B., Rodriguez-Galán M. (2015). Development of fly ash boards with thermal, acoustic and fire insulation properties. Waste Manag..

[36] (2021). Fire Resistance Tests Part 1: General Requirements.

[37] (2012). Gypsum Blocks Definitions, Requirements and Test Methods.

[38] (2019). Methods of Test for Mortar for Masonry—Part 11: Determination of Flexural and Compressive Strength of Hardened Mortar.

[39] (2006). Environmental Management—Life Cycle Assessment—Principles and Framework.

[40] Sonderegger T., Stoikou N. (2023). Implementation of Life Cycle Impact Assessment Methods in the Ecoinvent Database v3.10.

[41] Bušatlić I., Bušatlić N., Merdić N., Haračić N. Material and energy balance of production of gypsum. Proceedings of the 13th International Research/Expert Conference “Trends in the Development of Machinery and Associated Technology” TMT.

[42] Fundación Labein. Guía Técnica para la Medición, Estimación y Cálculo de las Emisiones al aire. Ed. *Soc. Pública De Gestión Ambiental del Gobobierno Vasco.* 2005. https://www.euskadi.eus/contenidos/documentacion/eprtr/es_guia/adjuntos/residuos.pdf.

[43] Gencel O., Díaz J.J., Sutcu M., Koksal F., Rabanal F.A., Martinez-Barrera G., Brostow W. (2014). Properties of gypsum composites containing vermiculite and polypropylene fibers: Numerical and experimental results. Energy Build.

[44] (2000). Replacing Decision 94/3/EC Establishing a List of Wastes Pursuant to Article 1(a) of Council Directive 75/442/EEC on Waste and Council Decision 94/904/EC Establishing a List of Hazardous Waste Pursuant to Article 1(4) of Council Directive 91/689/EEC on Hazardous Waste.

[45] Mercante I., Bovea M., Ibáñez-Forés V., Arena A. (2012). Life cycle assessment of construction and demolition waste management systems: A Spanish case of study. Int. J. LCA.

[46] (2024). Google Maps. Distances. https://www.google.com/maps/.

[47] (2007). On Type Approval of Motor Vehicles with Respect to Emissions from Light Passenger and Commercial Vehicles (Euro 5 and Euro 6) and on Access to Vehicle Repair and Maintenance Information.

[48] Goedkoop M., Oele M., Leijting J., Ponsioen T., Meijer E. Introduction to LCA with SimaPro. Ed. PRE Consultants, 2016. https://www.pre-sustainability.com/download/SimaPro8IntroductionToLCA.pdf.

[49] Huijbregts M.A.J., Steinmann Z.J.N., Elshout P.M.F., Stam G., Verones F., Vieira M.D.M., Zijp M., Van Zelm R. (2017). ReCiPe 2016 v1.1 A harmonized life cycle impact assessment method at midpoint and endpoint level Report I: Characterization. NIPHE (RIVM) Int. J. Life Cycle Assess.

[50] Salazar P.A., Fernández C.L., Luna-Galiano Y., Sánchez R.V., Fernández-Pereira C. (2022). Physical, Mechanical and Radiological Characteristics of a Fly Ash Geopolymer Incorporating Titanium Dioxide Waste as Passive Fire Insulating Material in Steel Structures. Materials.

[51] Leiva C., Gómez-Barea A., Vilches L.F., Ollero P., Vale J., Fernández-Pereira C. (2007). Use of biomass gasification fly ash in lightweight plasterboard. Energy Fuels.

