# Supplementary material for: Study of a Fire-Resistant Plate Containing Fly Ashes Generated from Municipal Waste Incinerator: Fire and Mechanical Characteristics and Environmental Life Cycle Assessment
Source: Materials (Basel). 2024 Apr 15;17(8):1813. doi: 10.3390/ma17081813 (PMC11050783; doi:10.3390/ma17081813)

# Supporting Information:

# Study of a Fire Resistance Plate Containing Fly Ashes Generated from Municipal Waste Incinerator: Fire and Mechanical Characteristics and Environmental Life Cycle Assessment

**Begoña Peceño [1], Yolanda Luna-Galiano [2], Fabiola Varela [1], Bernabé Alonso-Fariñas [2] and Carlos Leiva [2*]**

**Figure S1.** The environmental impact of the distance from gypsum calcination to production. Refer to Figure 6 for details on the nomenclature's significance.

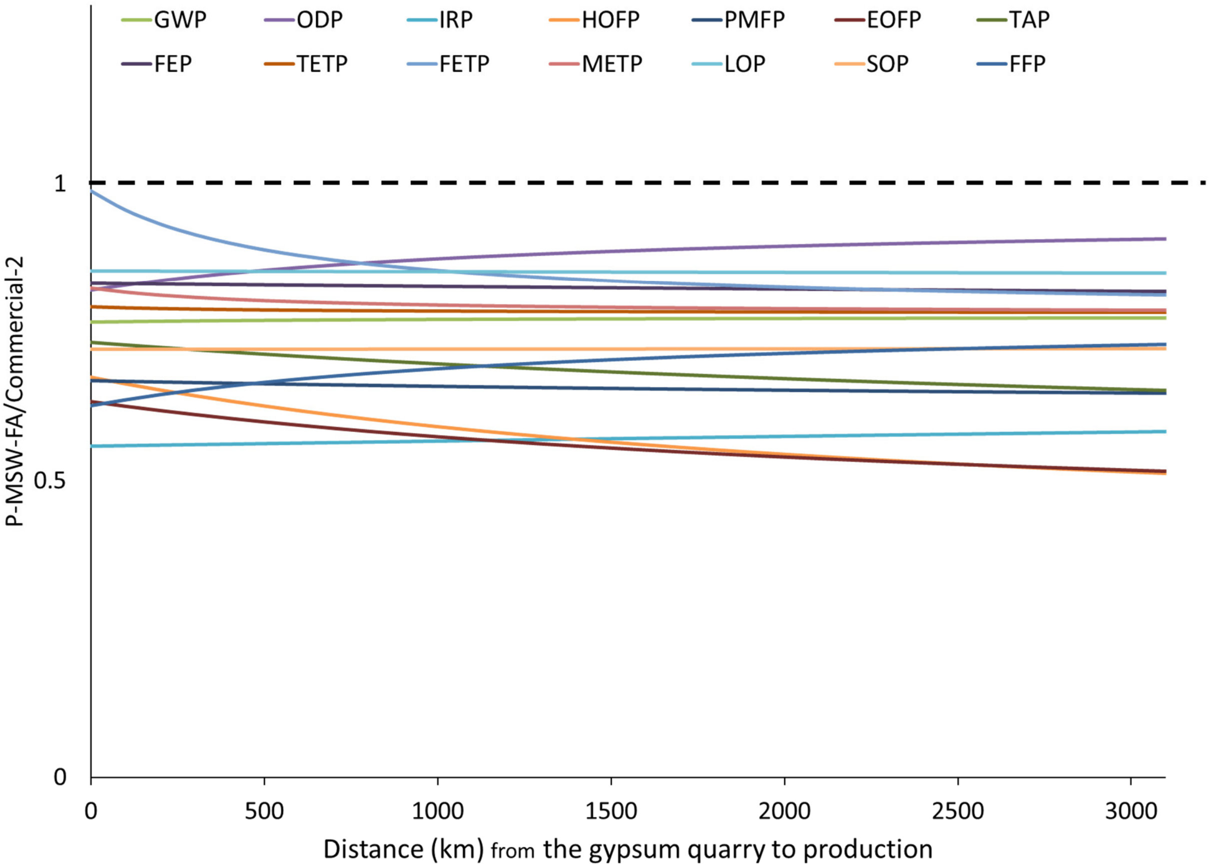

**Figure S2.** The environmental impact of the distance from vermiculite plant to production. Refer to Figure 6 for details on the nomenclature's significance.

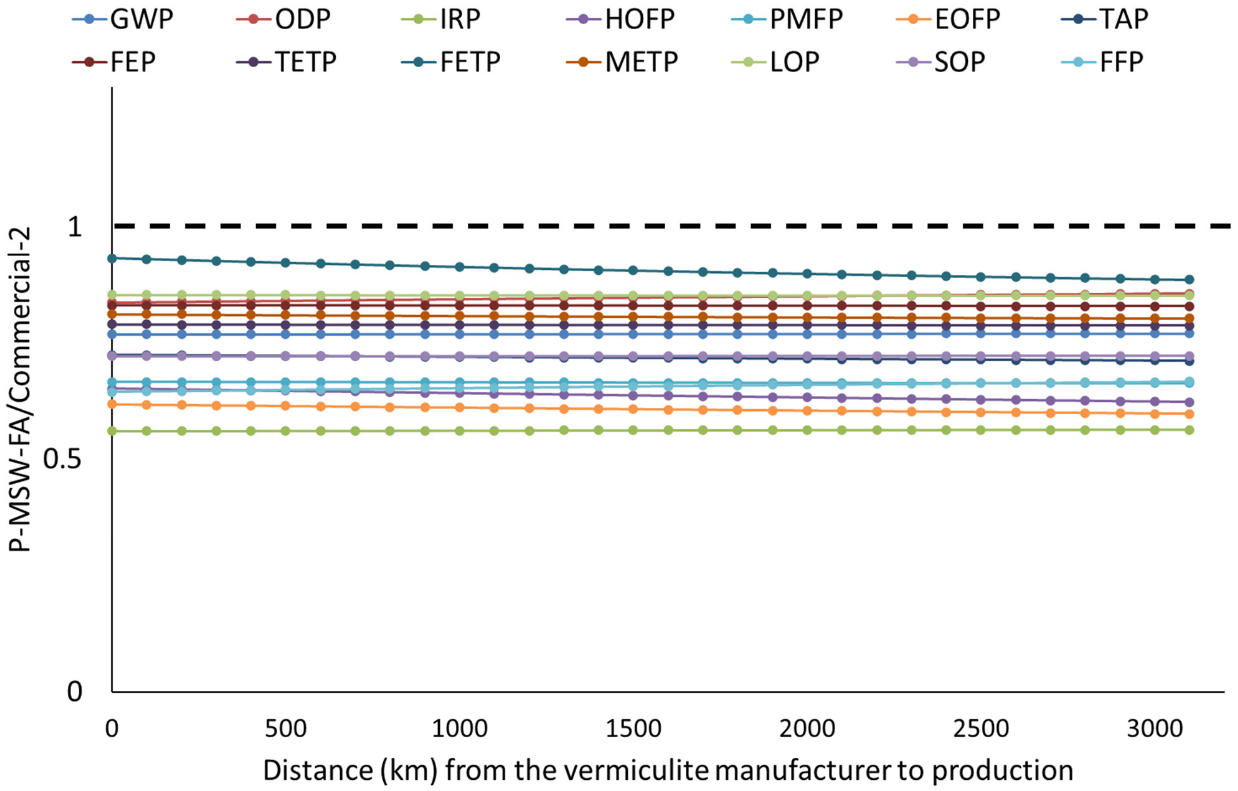

**Figure S3.** The environmental impact of the distance from glass fiber plant to production. Refer to Figure 6 for details on the nomenclature's significance.

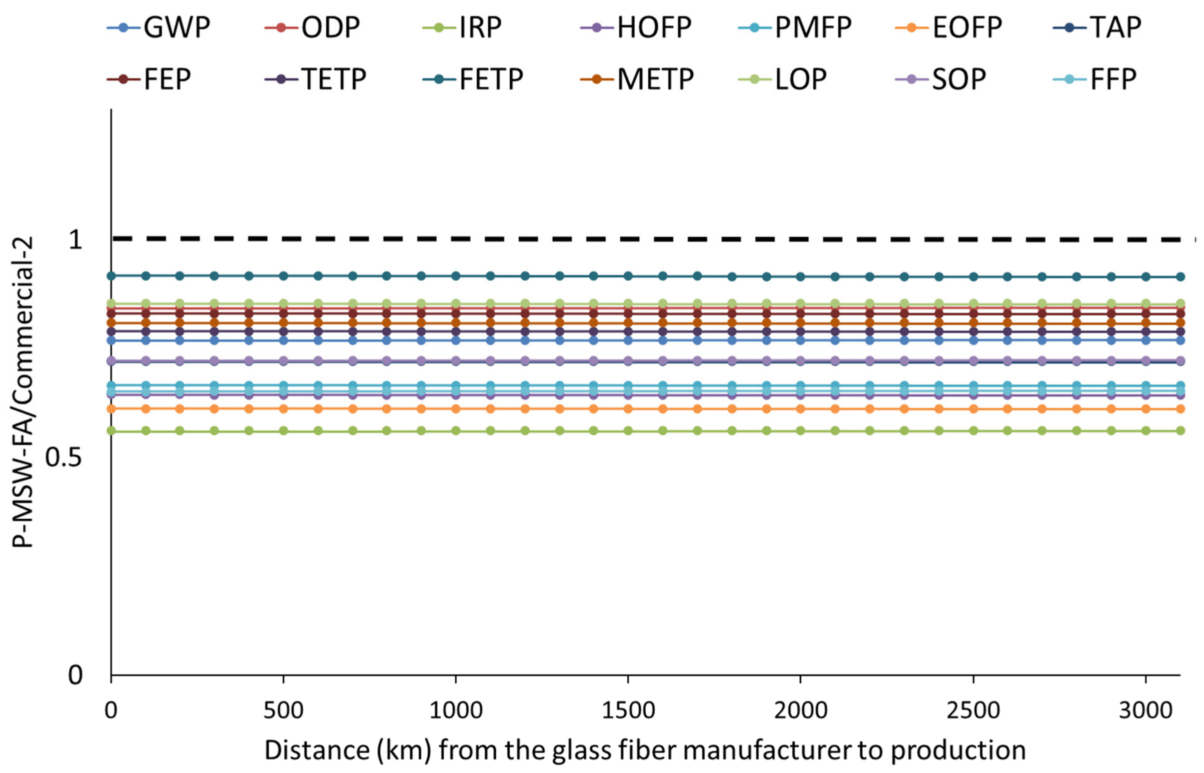

Supplement: Supplementary file 1 [file materials-17-01813-s001.zip › materials-2933861-supplementary.pdf]
